# Oral health in adolescents: periodontal inflammatory biomarkers during orthodontic clear aligner therapy

**DOI:** 10.1007/s00784-025-06212-9

**Published:** 2025-03-05

**Authors:** Carolin Kredig, Eva Peuckert, Irene Schmidtmann, Thomas Drechsler, Christina Erbe

**Affiliations:** 1 Department of Orthodontics, University Medical Center at the Johannes Gutenberg-University, Mainz, Germany; 2 Institute of Medical Biostatistics, Epidemiology and Informatics, Johannes Gutenberg-University Medical Center, Mainz, Germany; 3 Private Clinic Wiesbaden, Wiesbaden, Germany

**Keywords:** Inflammatory biomarker, Aligner therapy, Orthodontics, Matrix metalloproteinase-8, IL-1 polymorphism

## Abstract

**Objectives:**

This prospective study aimed to evaluate periodontal inflammation in adolescents undergoing orthodontic treatment with clear aligners (Invisalign® Teen, Align Technology, San Jose, CA, USA). Key objectives included assessing the presence of 11 periodontitis-associated marker bacteria, active matrix metalloproteinase-8 concentrations in sulcular fluid, and the influence of IL-1 polymorphism genotypes on periodontal health.

**Materials and methods:**

Fifty adolescent patients (13.3 ± 1.8 years) with mixed and permanent dentition participated. Gingival crevicular fluid samples were analyzed at multiple time points: before, during, and one year after aligner treatment. Periodontal health was assessed using the Gingivitis Index and the Modified Quigley-Hein Index. Genotypic analysis of IL-1 polymorphism was also performed. Statistical analyses included mixed linear models and generalized linear models to explore correlations.

**Results:**

All combinations of IL-1 polymorphism genotypes were found in the sample. No significant increase in periodontal inflammation or aMMP-8 concentrations was observed over the treatment period. Marker bacteria from the red and orange-associated complexes remained at low levels, while significant changes occurred in the orange and green complexes, particularly Capnocytophaga spp. (p = 0.0042) and Fusobacterium spp. (p = 0.0365). GI correlated significantly with aMMP-8 levels (p = 0.0017), but no genotype effect on GI was observed. MQH showed associations with pathogens from the orange and green complexes, including Capnocytophaga spp. and Fusobacterium spp.

**Conclusions:**

Clear aligner treatment in adolescents, including those with an unfavorable genotype, does not increase periodontal inflammation when accompanied by good oral hygiene.

**Clinical relevance:**

Regular periodontal monitoring and hygiene reinforcement is important during orthodontic treatment, especially in adolescent patients.

## Introduction

**Fig. 1:**
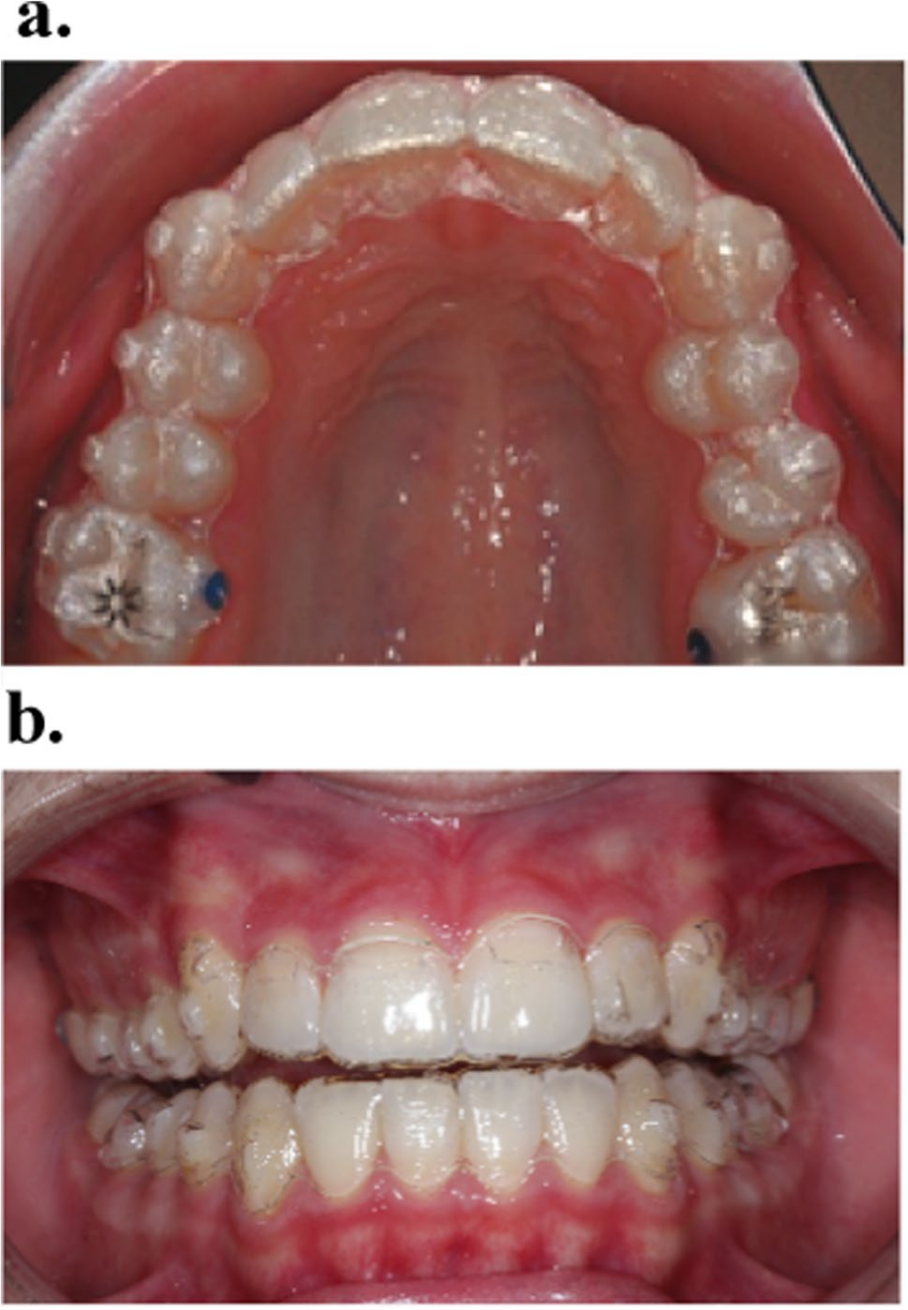
**a**. An example of clear aligner use in the treatment of crowding. **b**. An example of the scalloped trimline design

Initially, clear aligner therapy with Invisalign® (Align Technology, San Jose, CA, USA) was limited to adult patients in the permanent dentition [1]. However, in 2008, the introduction of Invisalign® Teen (Align Technology, San Jose, CA, USA) has extended this spectrum to adolescents, increasing the group of patients who can be treated with clear aligners considerably [2–6]. Furthermore, it is now possible to treat more and more complex cases with clear aligners, though closing extraction spaces remains one of the most challenging tasks. The recommended wear time for clear aligners is 22 h daily, covering both teeth and the gingival margin. This is raising concerns about periodontal inflammation [2–6]. Compared to fixed appliances, clear aligner therapy offers the notable advantage of simplifying daily oral hygiene through its removable design, while maintaining precise three-dimensional control of tooth movements (Fig. 1a) [7–9].

Poor oral hygiene and hormonal changes can also negatively affect oral health and contribute to various forms of gingivitis and periodontitis in adolescents [10]. Fortunately, chronic periodontal diseases are less prevalent in this age group compared to adult patients [10]. In Germany, the mandatory KIG system (Kieferorthopädische Indikationsgruppen, or Orthodontic Indication Groups) provides a standardized method for classifying the severity of malocclusion, which directly influences the eligibility for public health insurance coverage of orthodontic treatment. Under this system, treatment is generally initiated during the mixed dentition phase and progresses through the permanent dentition [11]. Therefore periodontal diagnostics and, if necessary, therapy should be carried out in adolescents undergoing orthodontic treatment in order to detect the onset of inflammation at an early stage and to prevent negative microbial dynamics leading to serious periodontal diseases 32 [12, 13].

The management of risk factors is considered an essential component in the prevention and treatment of periodontal disease. Major individual risk indicators with varying levels of evidence include age, gender, smoking, diabetes, obesity, osteoporosis, stress and genetic factors [14, 15]. Among these, smoking and diabetes are regarded as well-established risk factors for peridodontal disease [15]. Conversely, the presence of periodontal pathogens, such as Porphyromonas gingivalis, Tannerella forsythia and Aggregatibacter actinomycetemcomitans, may be classified as risk indicators rather than risk factors [13, 15].

### Indices

Gingivitis counts as an early indication, which is induced by plaque accumulation [16]. Given that the prevalence of gingival bleeding and other periodontal issues in adolescents can be as high as 42%, it is important to assess periodontal risk factors before starting orthodontic treatment [17]. Fortunately, gingivitis is fully reversible after plaque removal [16]. Therefore, if necessary, appropriate therapy should be initiated, even in adolescents undergoing orthodontic treatment. Typically, various indices like GI (Gingivitis Index according to Löe and Silness), PBI (Papillary Bleeding Index) and SBI (Sulcus Bleeding Index) are used to determine gingival health. Plaque accumulation is determined using indices like API (Approximal Plaque Index), TMQH (Turesky modification of the Quigley-Hein index) and O´Leary Index [18]. The GI is a widely used indicator for inflammation in research, facilitating comparability [14]. In addition, elevated levels of aMMP-8 and the presence of specific periodontal marker pathogens have been identified as potential indicators [19]. In addition, the relationship to orthodontic treatment is still unexplored. Limited research has yet focused on periodontal marker pathogens in association with orthodontic clear aligner treatment [3, 20, 21].

### Periodontal pathogens

Limited research has so far focused on periodontal marker pathogens in association with orthodontic clear aligner treatment [3, 20, 21]. The pathogens associated with periodontitis development, including periodontal pathogens and periodontitis-associated microorganisms, are categorized into five main complexes: Among these, the “red complex” comprises gram-negative, anaerobic bacteria, such as Porphyromonas gingivalis (Pg) and Tannerella forsythia (Tf), which are primary contributors to aggressive and chronic periodontitis as well as peri-implantitis. Additionally, the spirochete Treponema denticola (Td), a key pathogen in necrotizing ulcerative gingivitis (NUG), is also part of this complex [22–25]. In the "orange complex" is Fusobacterium spp. (Fs), which is one of several marker germs in chronic periodontitis, Prevotella intermedia (Pi) which is associated in different forms of Periodontitis and Parvimonas micra (Pm), a relatively rare germ that can be detected somewhat more frequently in advanced periodontitis. The "orange-associated complex" comprises the gram-positive anaerobic rods Campylobacter rectus (Cr) and Eubacterium nodatum (En) [23, 25]. The gram-negative facultative anaerobic rods Eikenella corrodens (Ec) and Capnocytophaga spp. (Cs) belong to the "green complex" [23, 25]. Eikenella corrodens (Ec) is regarded as a plaque-forming and recurrent pathogen. The Gram-negative, facultatively anaerobic rod Aggregatibacter actinomycetemcomitans (Aa) is distinct from these complexes [23, 25]. It is particularly associated with localized aggressive periodontitis (juvenile periodontitis) and destructive periodontitis in adults [22].

### Matrix metalloproteinase-8 (aMMP-8)

aMMP8, an endogenous enzyme, plays a major role in tissue destruction in periodontal diseases [26]. Its concentration of the active matrix metalloproteinase-8 (collagenase 2) indicates the host reaction to inflammatory events at an early stage. The importance of this concentration analysis has been described in various studies and is, therefore, a proven option in periodontal diagnostics, in addition to the microbiological examination of marker bacteria [27, 28]. In patients with periodontal disease, the aMMP-8 values are significantly higher than in healthy control groups [26, 29]. However, association with clear aligner therapies in adolescents remains unexplored.

### IL-1 polymorphism and different genotypes

Various factors influence periodontal inflammatory reactions, including the rate of development and the extent of the response [27, 28]. The body's immune response to the inflammatory process is genetically predisposed. This can be illustrated by the genotypes derived from the analysis of the IL-1 polymorphism [28, 30, 31]. Table 1 shows the relationship between the IL-1 polymorphism and the various genotypes. Genotypes derived from IL-1 polymorphism, particularly variants 2 and 3, are of interest concerning periodontal disease risk. An increased risk of periimplantitis has only been reported for genotype 4. The relationship between this IL-1 polymorphism and clear aligner therapy in adolescent patients has not yet been investigated.

**Table 1:** Explanation of the relationship between IL-1 polymorphism and the different genotypes: IL-1 polymorphism = Interleukin 1 Polymorphism; IL-1RN = Interleukin 1 receptor antagonist

Genotypes	IL-1	IL-1RN	Risk of inflammation	Periodontitis risk for progressive periodontits
1	normal	normal	normal	normal
2	increased	normal	increased	increased
3	increased	decreased	significantly increased	significantly increased
4	normal	decreased	decreased	frequent occurrence of periimplantitis

The aim of this clinical study, approved by the Ethics Committee of the Medical Chamber of Rhineland-Palatinate (9565), is to assess periodontal inflammation in adolescent patients by analyzing the 11 periodontitis-associated marker bacteria and aMMP-8 concentration in sulcular fluid before, during, and after aligner treatment. This clinical study focuses specifically on aspects of periodontal inflammation. In addition, the genetic disposition of adolescent patients was also considered by determining the IL-1 polymorphism.

## Materials and methods

This prospective study included 53 patients scheduled to undergo treatment with clear aligners (Invisalign® Teen, Align Technology, San Jose, CA, USA) in both the upper and lower jaws. Data collected between May 2015 and December 2023 were analyzed. All patients received treatment at the Department of Orthodontics and Dentofacial Orthopedics at the University Medical Center of the Johannes Gutenberg-University Mainz.

The sample size was planned based on a paired t-test. A sample size of n = 52 is required to establish a medium effect of d = 0.4 with 80% power at the 5% significance level. Assuming a 5% dropout rate, 55 patients were included.

A total of 53 patients initially consented to participate. Later, three were excluded for missing more than half of the visits. Among the remaining 50 patients, the genotype was not possible for two patients. And two additional samples could not be analyzed in the laboratory because the PCR was inhibited. Some patients have not yet completed all visits because of the long treatment duration.

Since patients were examined before, during and one year after orthodontic treatment with clear aligners, a separate control group was deemed unnecessary. The test subjects, therefore, served as their own control group during the phases without orthodontic treatment.

Inclusion criteria were the following:

- patients aged between 11 and 17 years
- planned treatment with Invisalign® Teen (Align Technology, San Jose, CA, USA)
- no previous treatment with fixed orthodontic appliances
- good systemic health, determined on the basis of a medical history survey
- a minimum of 16 permanent teeth, including eight anterior teeth with accessible facial and lingual surfaces
- maintenance of previous brushing habits.

Exclusion criteria were the following:

- syndromes or general diseases
- pregnancy
- more than three carious lesions
- severe periodontal disease, characterized by purulent exudates, tooth mobility, and/or gingival recessions
- use of antibiotics at the beginning and during treatment
- additional professional dental cleaning during treatment or changes to previous brushing habits

Patients were instructed to continue their usual brushing technique using a manual or an electric toothbrush. Brushing instructions were given at the beginning of the clinical study. Diet and the use of adjunctive therapies (e.g. mouthwash) were not permitted. On the days of examination, no dental care was permitted for at least 24 h prior.

The aligners were trimmed at the level of the enamel-cement junction with a scalloped edge (Fig. 1b). Patients were instructed to wear the clear aligners for at least 22 h per day. A typical 7-wear-protocol per aligner applied. They were cleaned under running water and, if necessary, brushed with mild toothpaste.

**Fig. 2:**
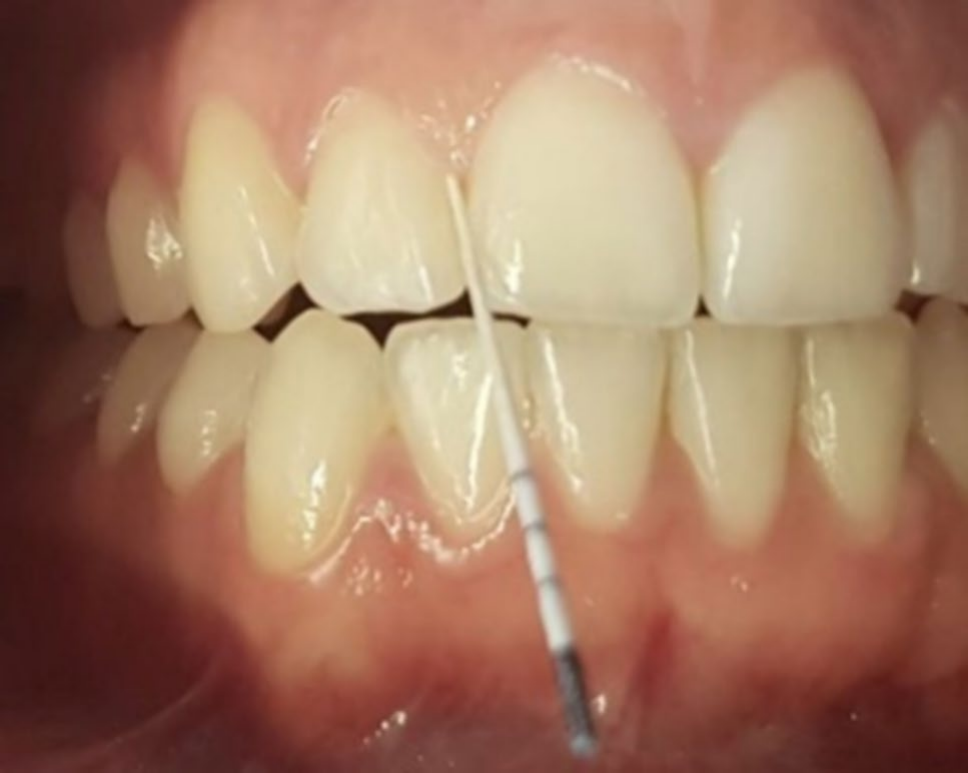
Example of periodontal pathogen examination with paper points in the mesial approximal space

**Fig. 3:**
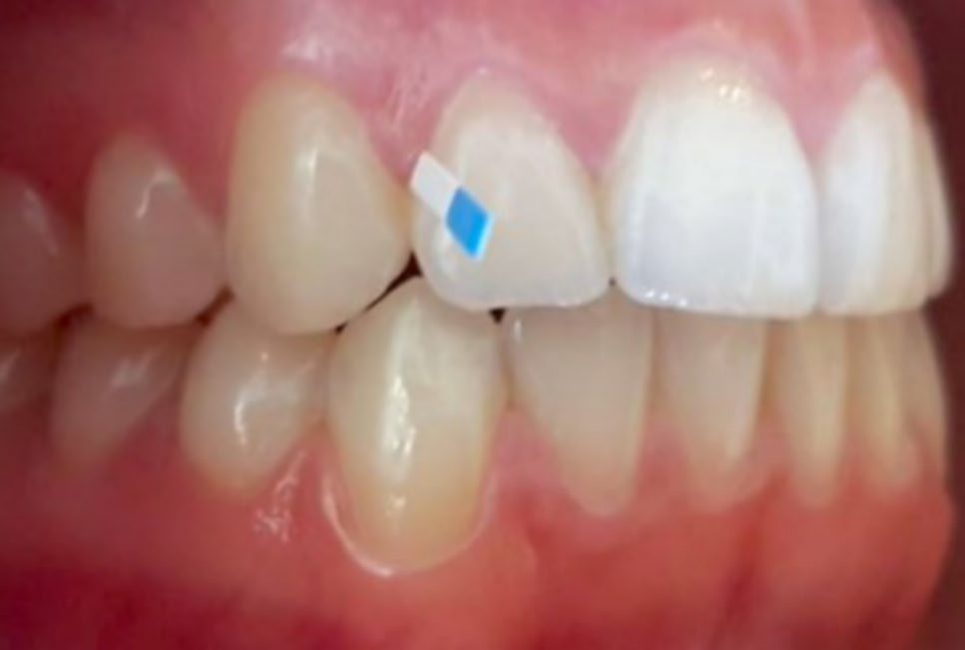
Example of sampling to determine the concentration of aMMP-8 with paper strips in the distal proximal space

The study´s protocol is outlined in Table 2. Informed consent was obtained from all study participants prior to commencing the study. All laboratory tests were conducted at the Bioscientia Laboratory (Institut für Medizinische Diagnostik GmbH, Berlin, Germany). Gingival sulcus fluid (GCF sample) was collected from Ramfjord teeth (16, 12, 24, 36, 32, and 44) [32]. GCF pool samples for determining the marker germs of the 11 periodontitis germs were collected from the mesial gingival sulcus using a paper tip for 30 s (Fig. 2), while pool samples for determining aMMP-8 were collected from the distal gingival sulcus using paper strips (Fig. 3). Since sampling time was consistently 30 s, as it is often the case in studies with GCF analyses, the concentrations in this study are given in ng/ml. The pool samples were taken for maxilla and mandible [33]. To analyze the 11 periodontitis-associated marker bacteria, nucleic acids were extracted from bacterial DNA (QiAamp DNA Mini Kit (QIAGEN N.V., Hilden, Germany) obtained from the GCF sample. The DNA-based in-vitro detection system LCD Array Kit BAC-Dent 2.4 (version: BAC-Dent 2.4 CE V-7.0–2013-GER, Chipron GmbH, Berlin, Germany) was employed for qualitative analysis (Chipron GmbH 2013). The aMMP-8 concentration in the sulcus fluid was determined using the "dentoELISA aMMP-8" enzyme immunoassay (Dentognostics GmbH, Jena, Germany). At the initial examination date (V1), an additional oral mucosa swab was collected using a cotton swab to determine the IL-1 polymorphism (Hain Lifescience GmbH, Nehren, Germany).

**Table 2:** Study procedure: Different procedures undertaken at different visits: GCF = Gingival crevicular fluid; IL-1 polymorphism = Interleukin 1 Polymorphism; GI Index = Löe-Silness Gingivitis Index; MQH = modifizierter Quigley-Hein-Index nach Kossak and Brinkmann

Procedure	Progress control	Professional tooth cleaning	GCF Sample	IL-1 Polymorphism	Oral hygiene instruction
Visit 1 Screening / Baseline			x	x	
Visit 2 Start of treatment 1 week after Screening	x	x			x
Visit 3 Inserting attachments 4 weeks after start	x	x	x		
Visit 4 6 months after start	x	x	x		
Visit 5 1 year after receiving the first aligner	x	x	x		
Visit 6 1 ½ years after start, removing of attachments	x	x	x		
Visit 7 1 year after end of therapy	x	x	x

The laboratory presented the results of the analysis of the 11 periodontitis-associated marker pathogens as follows:

- Germ not detected—(< 104 KbE)
- Low germ concentration ( +) (= 104 KbE)
- Increased germ concentration + (< 105 KbE)
- Highly increased germ concentration + + (< 106 KbE)
- Very high increased germ concentration + + + (> 107 KbE)

The determination of bacterial concentrations was standardized for all marker bacteria. However, for Aggregatibacter actinomycetemcomitans, the values were evaluated at one order of magnitude lower (e.g., + = 104 KbE).

The Gingival Index (GI) according to Löe-Silness was used to evaluate gingival color, consistency, inflammation, and bleeding upon probing. The assessment included the entire dentition, excluding the third molars. In both the maxilla and mandible, the gingival sulcus was examined using a periodontal probe, scanning from the buccal to the palatal aspect. The evaluation was categorized based on the following criteria: 0 = physiological, non-inflammatory gingiva; 1 = mild inflammation (slight color change, slight edema, no bleeding on probing); 2 = moderate inflammation (redness, edema and glaze, bleeding on probing); 3 = massive inflammation (marked redness, edema, ulcer, tendency to spontaneous bleeding) [34].

The modified Quigley Hein Index (MQH) according to Kossak and Jost-Brinkmann [35] was used to evaluate the plaque level on the vestibular tooth surface during clear aligner therapy. This modification is essential for these patients because, similar to multi-bracket appliances, attachments are present on the vestibular surfaces. The assessment is performed by staining the plaque in each quadrant individually using Mira-2-Ton dye (Hager & Werken GmbH & Co. KG, Duisburg, Germany) and evaluating three areas. Plaque accumulation in the distal, central, and mesial areas of the vestibular surfaces was classified to one of six categories between 0 (= no plaque) and 5 (= plaque extension more than two thirds of the tooth area) [35].

The collected data were compiled in Excel (Microsoft Office 2019 Home; Windows 10) and analyzed using the statistical software packages SAS 9.4 and R 4.4.1. The R package alluvial was specifically used to generate the alluvial plots [36].

A linear mixed model, with 'visit' as a fixed effect and 'patient' as a random effect was used to analyze the development of aMMP-8 concentrations over time. Changes in pathogen concentrations (given as ordinal categories) compared to baseline were assessed using the sign test. Linear mixed models were also used to evaluate the association of GI and TMQH with aMMP-8 and each pathogen marker, while accounting for genotype, visit, and upper/lower jaw. 'Patient' was again included as a random effect in these models.

In order to examine potential correlation between complexes and genotype, we dichotomized bacterial concentrations to bacterium present for each pathogen in lower and upper jaws, and fitted a generalized linear mixed model with genotype and visit as fixed effects, and patient as random effect. P-values less than or equal to 0.05 are termed significant. As the intention of the analysis is exploratory, no correction for multiple testing has been applied.

## Results

### Description of the patient cohort

A total of 27 boys and 23 girls were included in the study, with a mean age of 13.3 ± 1.8 years.

At the beginning of treatment, 13 deciduous teeth were present in six out of the 50 patients. Due to their position and the small number of erupted teeth, a separate analysis of aMMP-8 concentrations and the identification of marker pathogens in these deciduous teeth was not possible.

None of the patients had a positive family history of periodontal disease.

Not all parameters could be consistently recorded across all visits (V2-V7). Patient participation was discontinued in two cases after visit 5, and in 12 cases after visit 8.

### Gingivitis index according Löe-Silness (GI)

**Fig. 4:**
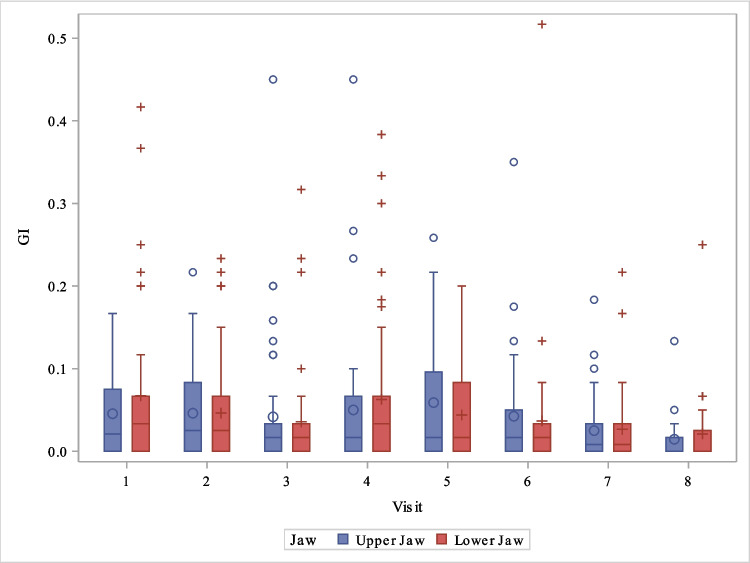
Boxplot distribution of gingival index as a function of upper jaw/lower jaw and visit

The distribution of gingival index as a function of upper/lower jaw and visit can be seen in Fig. 4.

### Modified Quigley Hein index (MQH) according to Kossak and Jost-Brinkmann

**Fig. 5:**
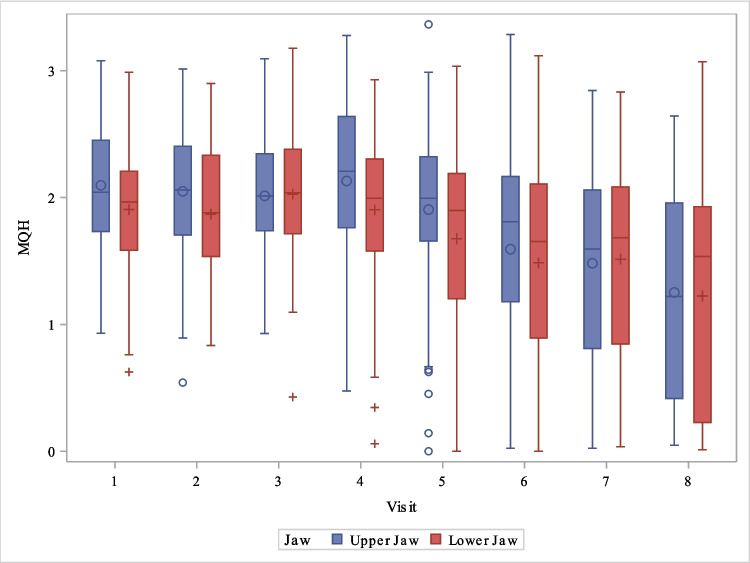
Boxplot Distribution of MQH as a function of upper jaw/lower jaw and visit

The distribution of MQH as a function of upper jaw/lower jaw and visit can be seen in Fig. 5.

### Frequency of IL-1 polymorphism genotypes

Genotype information was available in 48 cases, genotype 1 of the IL-1 polymorphism was observed in 12 cases within the analyzed patient population, while type 2 occurred 11 times. Genotypes 3 and 4, associated with a genetically higher risk of inflammation, were present in 4 cases (0 female/ 4 male) and 21 cases (11 female/ 10 male), respectively.

### Occurrence and concentration changes of marker pathogens during the course of treatment

**Fig. 6:**
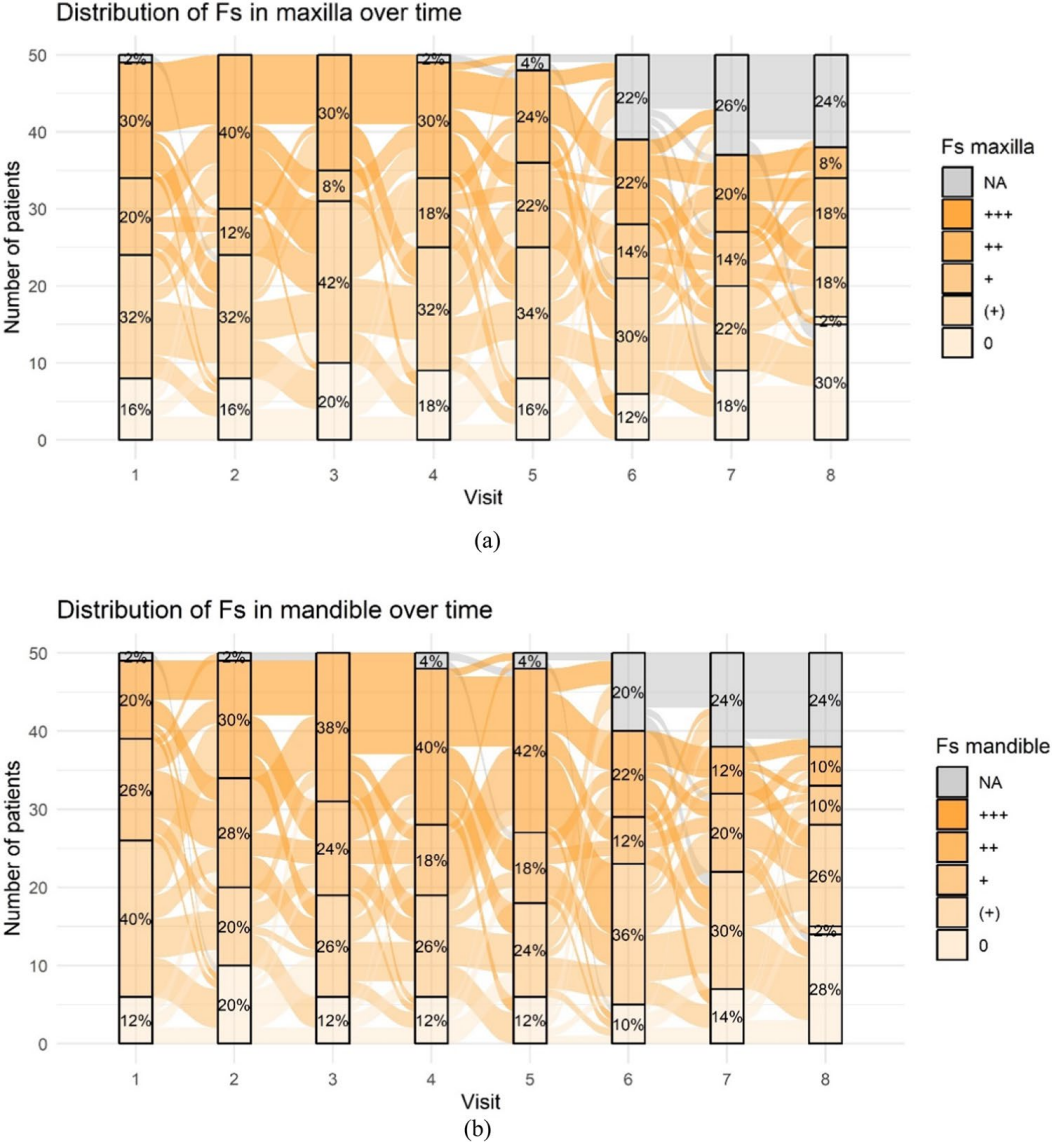

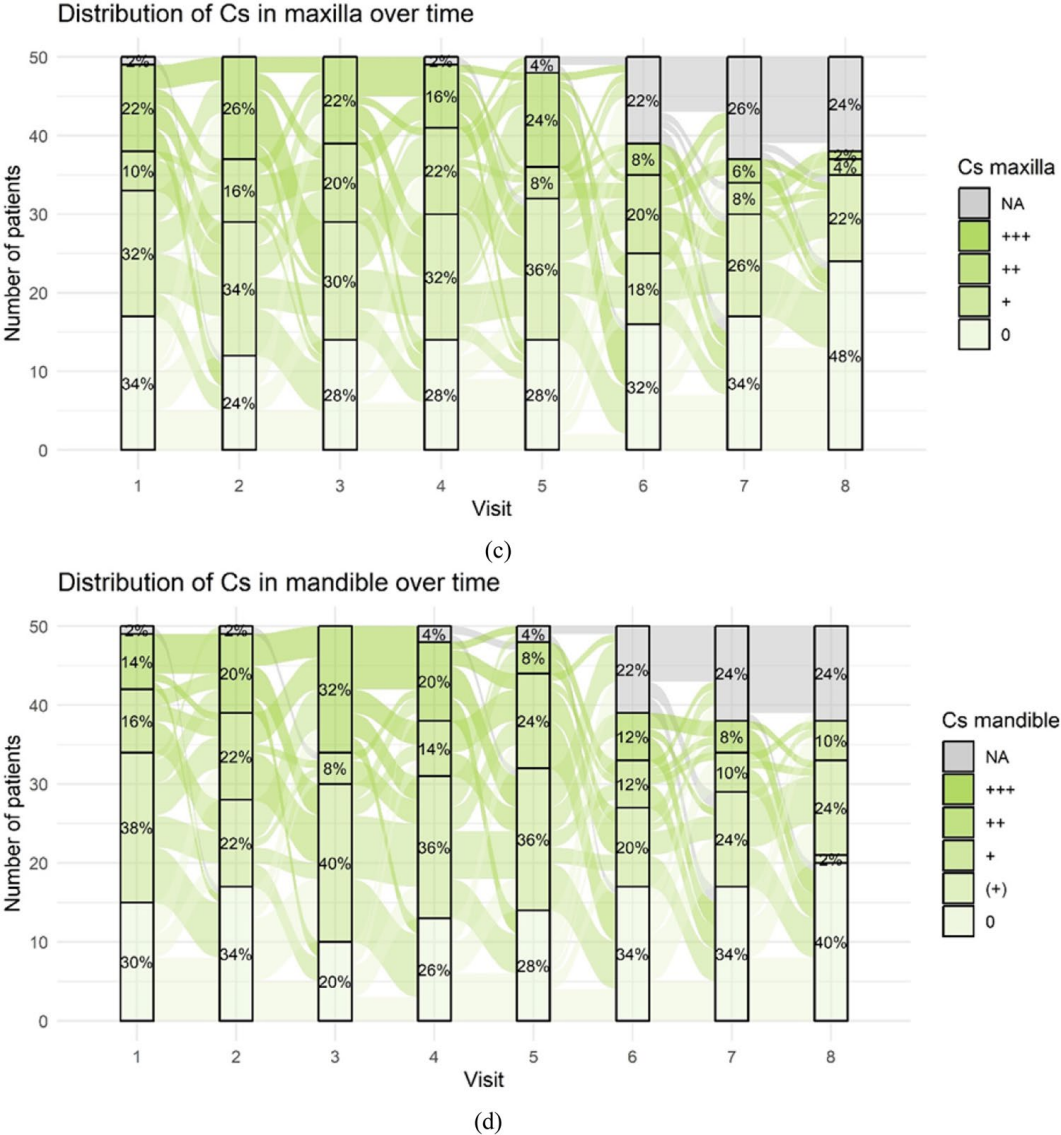
Frequency distribution of periodontitis marker bacteria complexes: (**a + b)** Frequency distribution of Fusobacterium spp. over time as an example of strong changes in the orange complex (**a**: maxilla, **b**: mandible); (**c + d**) Frequency distribution of Capnocytophaga spp. over time as an example of strong changes in the green complex (**c**: maxilla, **d**: mandible). The concentration of Fs and Cs is given as 0, ( +), + , + + , + + + representing no detection of the marker bacterium, low concentration, increased, strongly increased and very strongly in-creased levels respectively

The distribution of specific periodontitis marker bacteria complexes throughout the study period varied substantially depending on the type of pathogen. No or very low concentrations of pathogens were observed in the red and orange-associated complexes during the examination period. Significant changes were noted in the orange and green complexes only. Figure 6a and b present values of (Fs) Fusobacterium spp. for the orange complex. Figures 6c and d display values of (Cs) Capnocytophaga spp. for the green complex. (Aa) Aggregatibacter actinomycetemcomitans sporadically occurred in low concentrations in 1–2 patients during the study period (not shown).

### Changes in aMMP-8 concentration

**Fig. 7:**
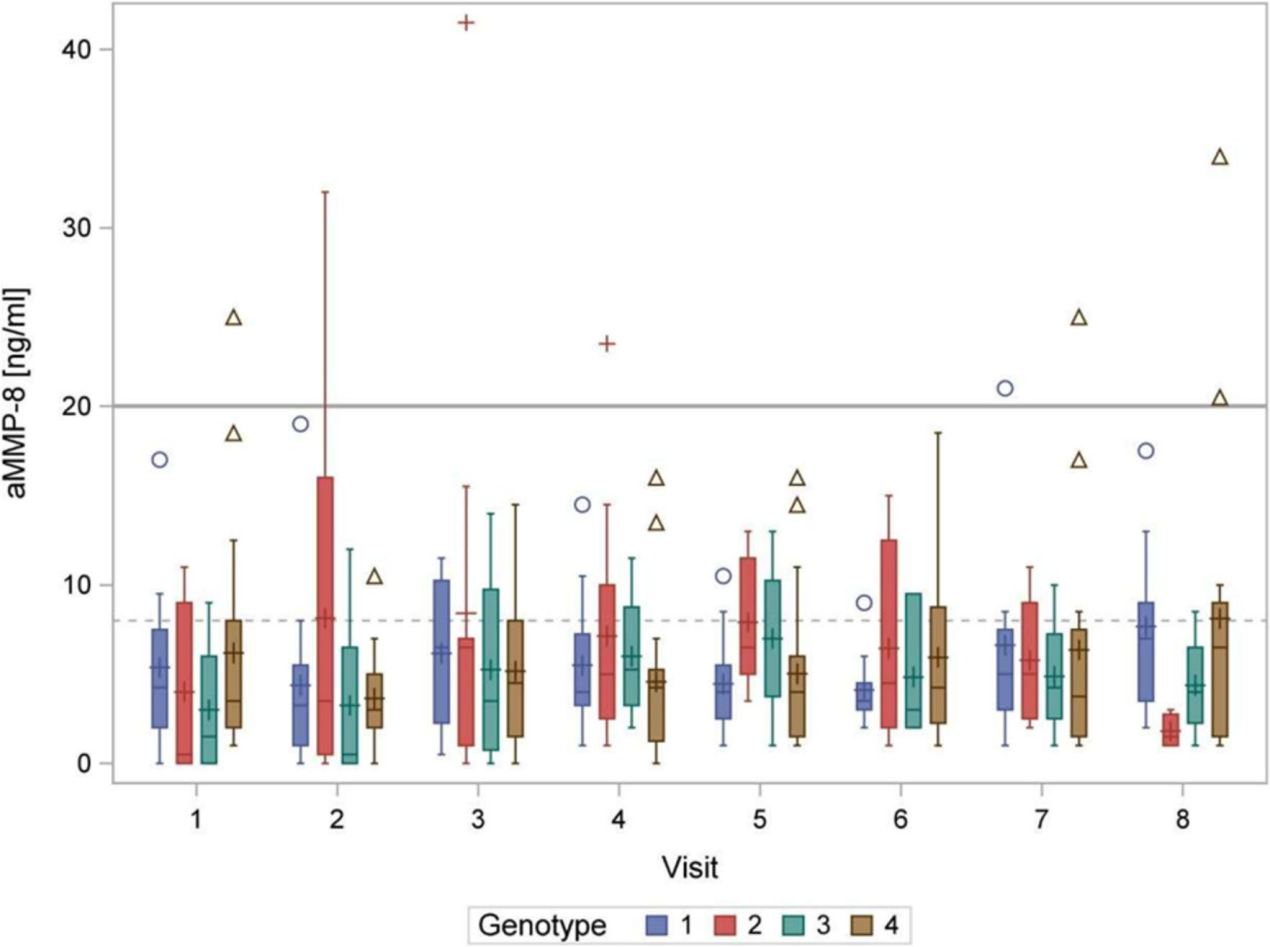
Boxplot of aMMP-8 concentrations [ng/ml] divided by genotype and by visits 1 to 8. The additional marker within the "box" represents the arithmetic mean. Values below ≤ 8 ng/ml (normal) indicate a very low risk of gingivitis), between 8 and 20 ng/ml (borderline) indicate low-grade local inflammation and ≥ 20 ng/ml (elevated) indicate acute inflammation [37]

The aMMP-8 concentration, averaged over maxilla and mandible, which indicates the tendency to inflammation, is depicted over time and stratified by genotypes in (Fig. 7). Statistical testing revealed no evidence of differences (p ≤ 0.05) in the aMMP-8 concentration over time or in the aMMP-8 concentration depending on the genotype or between the sexes (p = 0.273 to 0.904).

### Correlations

To assess potential correlations between complexes and genotype, we dichotomized the concentration of bacteria as present or absent in the upper or lower jaw, and fitted a generalized linear mixed model with genotype and visit as fixed effects, and patient as a random effect.

Some pathogens were too rare for the model to converge, making it impossible to draw any conclusions. This was the case for Aa, En, Pg, and Td. On the other hand, Fs was detected in the majority of patients, regardless of genotype. Therefore, no conclusions could be made regarding the correlation between genotype and pathogen occurrence.

For the remaining pathogens Cr, Cs, Ec, Fs, Pi, Pm and Tf, significant associations of genotype and presence only for Cs (p = 0.040) were found. Patients with genotype 3 were less likely to exhibit the presence of the pathogen. When assessing the association of (GI) Gingivitis Index Löe-Silness with aMMP-8 and marker pathogens and genotype, a significant association for GI and aMMP-8 (p = 0.0017) was found, but no effect of genotype on GI was observed (p = 0.3945). On average, GI increased by 0.0013 for each unit increase in aMMP-8.

Concerning the pathogen markers, no effects were found on GI of markers (Aa) Aggregatibacter Actinomycetemcomitans (p = 0.8579), (Cr) Campylobacter rectus (p = 0.5044), (En) Eubacterium nodatum (not estimable as too rare), (Pg) Porphyromonas gingivalis (p = 0.2734), (Pi) Prevotella intermedia (p = 0.3006), (Pm) Parvimonas micra (p = 0.4198), (Td) Treponema denticola (p = 0.1356) and (Tf) Tannerella forsythia (p = 0.8861). Significant associations of GI were observed with (Cs) Capnocytophaga spp. (p = 0.0042) and (Fs) Fusobacterium spp. (p = 0.0365). A slight, but non-significant association was observed with (Ec) Eikenella corrodens (p = 0.0690). On average, GI increased by 0.0043 for Cs + and by 0.0053 for Cs + + compared to non-detectable Cs. On average, GI increased by 0.0052 for Fs + , 0.0060 for Fs + + , and 0.0057 for Fs + + + , compared to non-detectable Fs.

When assessing the association between the Modified Quigley-Hein Index (MQH), aMMP-8, marker pathogens, and genotype, no significant correlation was found between MQH and aMMP-8 (p = 0.3255), nor was any effect of genotype on MQH observed (p = 0.9568).

Concerning the pathogen markers, no effects on MQH of markers Aa (p = 0.3696), Ec (p = 0.1646), Pg (p = 0.4727), Pi (p = 0.15819), Td (p = 0.4766) and Tf (p = 0.3920) were found. Significant associations of MQH were observed with Cr (p = 0.0011), Cs (p = 0.0017), Fs (p = < 0.0001) and Pm (p = 0.0024). On average, MQH increased by 0.055 for Cr + + and by 0.064 for Cr + + + compared to non-detectable Cr. On average, MQH increased by 0.4024 for Cs ( +) compared to Cs non detectable. On average, MQH increased by 0.2940 for Fs ( +), by 0.0527 for Fs + + + compared to Fs non-detectable. On average, MQH increased by 0.0626 for Pm + , by 0.2034 for Pm + + + compared to Pm non detectable.

## Discussion

### Main findings

All combinations of IL-1 polymorphism genotypes were found in the sample. No significant change in aMMP-8 concentration was observed over time of treatment. The distribution of specific periodontitis marker bacteria varied, depending on the type of pathogen. Significant changes were especially noted in the orange and green complexes. There is no indication of a correlation between genetic disposition and level of aMMP-8 concentration. Significant correlations (p < 0.05) were found when examining the relationships between GI and MQH on the one hand, and aMMP-8, genotype, and marker pathogens on the other hand. These correlations were observed between GI and aMMP-8, as well as between MQH and the marker pathogens Cr, Cs, Fs, and Pm.

### Methodological discussion

The patient cohort analyzed in this study consists of periodontal healthy adolescent. This was ensured by defining known influencing factors of periodontal diseases, such as general diseases, syndromes, and pregnancy, as exclusion criteria [12, 26]. To ensure that the effects of clear aligner treatment on periodontal health were not overshadowed by individual variations in dental hygiene, professional tooth cleaning was performed after each visit. Unfortunately, due to the long treatment time and partially limited patient compliance during the Covid-19 pandemic, not all parameters could be consistently recorded at all time points V2-V7. From visit 5 onwards the values of 2 (visit 5) to 12 (visit 8) patients had discontinued study participation. Moreover, the genotype result was not communicated by the laboratory for two patients. The aMMP-8 concentration in the sulcus fluid was determined using the "dentoELISA aMMP-8" enzyme immunoassay. As similar changes could be seen in all patients, the few missing data can be neglected. As already previously mentioned a sample size calculation was performed and n = 52 is needed to establish a medium effect of d = 0.4 with 80% power at the 5% significance level. Assuming a 5% dropout, 55 patients were initially included. Due to COVID-19, only 50 patients could be recruited, several of whom did not participate in all follow-up visits.

Since the cost of aligner treatment is not covered by public health insurance in Germany, patients must pay for this therapy privately. Consequently, there may be a slight bias in the study, as patients who are financially invested in their treatment may also be more diligent about maintaining their oral hygiene and more motivated achieve a successful treatment outcome.

### Plaque und gingivital indices

While increased plaque accumulation with subsequent gingival inflammation is commonly observed during fixed orthodontic treatment [38, 39], no general significant increase in the corresponding values was found during treatment with clear aligners. Isolated outliers, as seen in Figs. 4 and 5, occurred in the adolescent patient group. This is not surprising, as the patients were in a transitional developmental phase (puberty), often characterized by low motivation and a lack of enthusiasm.

### Genotype IL-1 polymorphism

Several studies have confirmed that the risk of developing periodontitis is genetically determined and significantly associated with IL-1 polymorphism [27, 40]. The distribution of IL-1 polymorphism genotypes in the analyzed cohorts is relatively even, encompassing all possible genetic predispositions. No signs of a correlation between genetic disposition and the level of aMMP-8 concentration were found, as all different genotypes showed normal (**≤ **8 ng/ml) or borderline (8–20 ng/ml) concentrations of aMMP-8 at most visits. This suggests that even patients with an unfavorable genotype do not necessarily experience a worsening of their periodontal situation during orthodontic treatment with clear aligners. Routine dental cleanings and oral hygiene instructions may account for these findings.

### Marker pathogens

In addition to the adsorption on paper points used here, bacteria can also be extracted from the sulcus by curettage [41]. These two methods lead to different results, as curettage extracts the entire plaque, whereas paper points are specifically used to collect tissue-damaging pathogenic bacteria from the outer layers of the biofilm [42]. Given the focus of this study on periodontal pathogens, the preferred method was collecting samples using paper points. Until now, there is a lack of studies on examining periodontitis-associated marker bacteria under aligner treatment in adolescent patients. Abbate et al. compared the periodontal health of adolescent patients under orthodontic treatment with clear aligners or fixed appliances. Patients treated with fixed appliances showed poorer hygiene and less periodontal health. Tests did not show the presence of anaerobic periodontal bacteria in any of the test subjects [3]. The bacterial concentration in the red and orange complexes in adult patients undergoing orthodontic treatment with clear aligners or fixed appliances varies across different studies. Sfondrini et al. and Lombardo et al. [43, 44] did not find significant changes in the proportions of red and orange complexes, whereas several other authors [45–49] report a higher burden of marker germs from the red complex species, C rectus and F nucleatum. This is in line with our results showing significant changes for the orange and green complexes. Additionally, Gujar et al. [50] describe a higher concentration of these complexes in patients undergoing orthodontic treatment, although the concentration was notably lower during clear aligner therapy. The examined pathogens are not causing periodontal diseases but are more important for caries development. However, the lower bacterial load when using clear aligners, as compared to fixed appliances, is also evident here [50]. This contrasts with studies investigating changes in periodontal marker germs during orthodontic treatment with fixed appliances. Hereby, the subgingival ecosystem underwent significant changes following the insertion of the appliances, leading to an increase in concentration of periodontal marker germs [51]. Bands extending into the subgingival region and the resulting increase in plaque accumulation [52] help to explain this phenomenon. Over the course of treatments, bacterial levels decreased again, and no increased risk of destructive periodontal diseases was found. Nevertheless, it is important to emphasize intensive oral hygiene instruction, remotivation, and monitoring during orthodontic treatment [51–54]. In case of periodontal diseases, which are fortunately rather rare in adolescent patients, the use of orthodontic bands should be avoided if possible [55].

### aMMP-8 concentration

The concentration of aMMP-8 in the sulcus fluid is generally regarded as a good indicator of periodontitis [56]. In their study, Ingman et al. found that although the concentration of aMMP-8 increased during orthodontic treatment with a fixed appliance, the values were lower than in periodontitis [39, 57]. Various studies have shown that tooth movement also leads to an increase in the concentration of aMMP-8 [58, 59]. This means that orthodontic treatment always involves paying particular attention to oral hygiene to minimize the dual problem (difficult oral hygiene and tooth movement) from the outset. The few studies that have investigated the change in aMMP-8 concentration during orthodontic treatment have either found an initial increase [57, 60] or no significant changes [59–61]. However, the studies were only conducted during the initial phase of orthodontic treatment for a maximum of one month, as it was assumed that the initial exertion of force to move the teeth influences the aMMP-8 concentration in the sulcus fluid. Peron et al. investigated the change in adolescents with fixed orthodontic appliances and found that the high concentration of aMMP-8 in the sulcus fluid remained constant over the entire period of treatment, indicating an increase in periodontal inflammation [39]. In the present study, it could now be shown that with good oral hygiene and appliances that are less susceptible to plaque (clear aligners), no significant change in aMMP-8 concentration was observed over the course of the entire treatment. aMMP-8 concentration also appears to be independent of the genotype of the IL-1 polymorphism, as no significant differences were found between the genotypes, which increase the risk of inflammation to varying degrees, nor were there any patients who developed periodontitis requiring treatment during orthodontic treatment. To summarize the aMMP-8 concentration, it can be said from the literature that this parameter, in contrast to the marker pathogens, is an important point for the observation of periodontal inflammation during orthodontic treatment due to its accuracy and low scattering. In our study aMMP-8 concentration did not show this great influence. Although a laboratory test is always required for determination, meaning that the parameter cannot be used for routine diagnostics during the course of treatment for time and financial reasons, the determination of the aMMP-8 concentration should no longer be omitted in future studies dealing with periodontal events during orthodontic treatment [29].

### Correlations

Examining the relationship between genotype and the presence of various periodontal pathogens, no significant correlations were found in the current patient population. However, in genotype 3, the marker pathogen Cs from the green complex appears to be detected significantly less frequently (p = 0.04).

Previous studies have reported a mild correlation between genotype and the increased prevalence of bacteria from the red and orange complexes [62, 63]. There appears to be a statistically significant correlation (p = 0.0017) between the level of GI and the value of aMMP-8.

On average, mean of GI increased 0.0013 categories per unit of aMMP-8. No correlation was found between GI and genotype. Regarding the correlation between GI and the different marker pathogens, a statistically significant correlation was observed only for the pathogens Cs (p = 0.0042) of the green complex and Fs of the orange complex (p = 0.0365). In the case of MQH, no correlations were found with aMMP-8 levels (p = 0.3325), genotype (p = 0.9568), or the pathogens of the red complex and Aa.

Striking correlations in MQH were observed for Cs from the green complex, as well as for Fs and Pm from the orange complex, and Cr from the orange-associated complex. To date, few studies have confirmed or refuted these specific correlations in the context of clear aligner treatment. Shokeen et al. [64] also report in their study on small changes in Pa risk indicator values and marker bacteria concentrations. Further studies would be necessary to not only examine the Pa risk factors, such as GI and MQH, or the change in the concentration of subgingival marker bacteria, but also to evaluate both complexes in larger numbers of patients, as it has been the case to date. The meaningful correlations that could not be determined in the present study, particularly regarding the period of orthodontic treatment, could be due to frequent professional tooth cleaning in this study. As Al-Mutairi et al. [65] describe the same mechanism: "orthodontic appliances complicate oral hygiene maintenance, leading to more plaque buildup and microbiological shifts, which can result in oral complications" [52].

### Periodontal health and invisalign®/invisalign® teen

Early studies aimed to prove the advantage of removable clear aligners over fixed appliances in orthodontic treatment regarding oral health [5, 8, 20, 21]. The removability of the clear aligners and the resulting unrestricted oral hygiene were repeatedly emphasized as positive factors [5, 8, 20, 66, 67]. However, these studies only paid general attention to periodontal health and/or the amount of biofilm mass. The marker bacteria, the aMMP-8 concentration, or the genotype was not determined. Meta-analyses published on comparing clear aligners and fixed appliances [68–70] and Systematic Review of Systematic Reviews [71] also failed to determine the parameters collected in the present study. The study by Rossini et al. even found a trend towards an improvement in the periodontal health indices [6, 72]; a finding that can also be seen to some extent in the present study. One explanation for this could be that poor oral hygiene causes plaque to accumulate on the inside of the aligners, leading to a discoloration of the aligners that is clearly visible to the patient [67]. Our results are in line with the study by Tuncay et al. who only investigated the gingival bleeding index during orthodontic treatment of adolescents with clear aligners (Invisalign® Teen), but also found no periodontal disease, but at most a slight tendency to bleed [2]. Other investigators even found a slight decrease in gingival bleeding tendency in the course of orthodontic aligner treatment [3, 20]. In our present study, as in Zhao et al. [73], no significant changes in the periodontal situation were observed. We suspect that this effect is partly due to the regular professional dental cleanings and oral hygiene instruction and therefore want to stress again the importance of ongoing oral hygiene monitoring during orthodontic treatment [7].

In this study we used a scalloped trimline at the juxtagingival rim. Faveo et al. examined the role of different trimlines concerning periodontal health [74]. In their adolescent patient group, they observed an increase in periodontal indices when a short trimline was used. Traversa et al. showed a positive effect on tooth movement, but less frication using a short trimline [75]. As the retention of the clear aligners must be improved by attachments with a short trimline, more retention niches are created for plaque accumulation. Considering the aforementioned results, it may be advisable to use a higher trimline in adolescent patients.

Comparing the effects of clear aligners and traditional removable appliances, Chen et al. [76] found no significant increase in risk indicators or indices between the two groups of appliances. They examined younger children (mean age 8–9 years) during the mean face of tooth eruptions. The missing worsening of indices of our study can be explained by the different age and dental status of the patient groups. In a systematic review by Chen et al. it was outlined that patients wearing removable orthodontic appliances showed significant increases for supragingival rods, subgingival Spirochetes, whereas Aggregatibacter actinomycetemcomitans (Aa) was nearly absent [77]. Anaerobic bacteria were detected in the subgingival dental plaque with the same density (n = 15.75%) at baseline and at three months, while the prevalence appeared increased, though not reaching statistical significance, at nine months (17.85%). The most important bacteria that cause periodontal tissue loss, as Aggregatibacter actinomycetemcomitans (Aa), Porphyromonas gingivalis (Pg), Tannerella forsythia (Tf), and Prevotella nigrescens, were not detected in any patients [78].

## Conclusions

Due to the remodeling of the bone and periodontal tissue in adolescents [12], close attention must be given to the periodontal marker pathogens, especially particularly in adolescent patients. To identify potential risks before destructive changes occur, the present study assessed the presence of 11 periodontitis-associated marker bacteria and the concentrations of active matrix metalloproteinase-8 (aMMP-8). The results revealed that periodontal inflammation does not increase in adolescents undergoing orthodontic clear aligner treatment with Invisalign® Teen (Align Technology, San Jose, CA, USA), highlighting the superiority of clear aligners over fixed appliance treatments in term of periodontal health [39]. Nevertheless, as prevention of negative microbial dynamics in oral health remains essential, maintaining excellent oral hygiene is crucial during orthodontic treatment. Additionally, regular professional cleanings and periodic hygiene instructions should be emphasized to support optimal oral health.

## Data Availability

Data will be made available on request.

## References

[1] Christensen GJ (2002) Orthodontics and the general practitioner. J Am Dent Assoc 133:369–71. 10.14219/jada.archive.2002.0178 11934193 10.14219/jada.archive.2002.0178

[2] Tuncay O, Bowman SJ, Amy B, Nicozisis J (2013) Aligner treatment in the teenage patient. J Clin Orthod 47:115–9; quiz 140 23660763

[3] Abbate GM, Caria MP, Montanari P, Mannu C, Orru G, Caprioglio A, Levrini L (2015) Periodontal health in teenagers treated with removable aligners and fixed orthodontic appliances. J Orofac Orthop 76:240–250. 10.1007/s00056-015-0285-5 25929710 10.1007/s00056-015-0285-5

[4] Azaripour A, Weusmann J, Mahmoodi B, Peppas D, Gerhold-Ay A, Van Noorden CJ, Willershausen B (2015) Braces versus Invisalign(R): gingival parameters and patients’ satisfaction during treatment: a cross-sectional study. BMC Oral Health 15:69. 10.1186/s12903-015-0060-4 26104387 10.1186/s12903-015-0060-4 PMC4478712

[5] Miethke RR, Vogt S (2005) A comparison of the periodontal health of patients during treatment with the Invisalign system and with fixed orthodontic appliances. J Orofac Orthop 66:219–229. 10.1007/s00056-005-0436-1 15959635 10.1007/s00056-005-0436-1

[6] Schaefer I, Braumann B (2010) Halitosis, oral health and quality of life during treatment with Invisalign((R)) and the effect of a low-dose chlorhexidine solution. J Orofac Orthop 71:430–441. 10.1007/s00056-010-1040-6 21082306 10.1007/s00056-010-1040-6

[7] Baumer C, Schmidtmann I, Ohlendorf D, Ferrari Peron P, Wehrbein H, Erbe C (2024) Orthodontists’ instructions for oral hygiene in patients with removable and fixed orthodontic appliances. Int J Dent Hyg 22:329–336. 10.1111/idh.12763 37845796 10.1111/idh.12763

[8] Miethke RR, Brauner K (2007) A Comparison of the periodontal health of patients during treatment with the Invisalign system and with fixed lingual appliances. J Orofac Orthop 68:223–231. 10.1007/s00056-007-0655-8 17522806 10.1007/s00056-007-0655-8

[9] Nedwed V, Miethke RR (2005) Motivation, acceptance and problems of invisalign patients. J Orofac Orthop 66:162–173. 10.1007/s00056-005-0429-0 15827703 10.1007/s00056-005-0429-0

[10] Bimstein E, Matsson L (1999) Growth and development considerations in the diagnosis of gingivitis and periodontitis in children. Pediatr Dent 21:186–191 10355010

[11] Seeling S, Prütz F (2018) Inanspruchnahme kieferorthopädischer Behandlung durch Kinder und Jugendliche in Deutschland – Querschnittergebnisse aus KiGGS Welle 2 und Trends. J Health Monit 3:. 10.17886/RKIGBE2018094

[12] Kasaj A, Willershausen B (2013) Periodontal diseases in children and adolescents. Monatsschrift Kinderheilkunde 161:518–523. 10.1007/s00112-012-2833-z

[13] Noack B (2017) Parodontale Erkrankungen bei Kindern und Jugendlichen. In: Zahnärzteblatt Sachsen 03/17. pp 23–26. https://www.zahnaerzte-in-sachsen.de/fileadmin/Publikationen/LZKS/IZZ/DOKUMENTE/ZBS/Fachbeitraege/2017/ZBS_03-2017_Parodontale_Erkrankungen_bei_Kindern_und_Jugendlichen.pdf

[14] Borgnakke WS, Ylostalo PV, Taylor GW, Genco RJ (2013) Effect of periodontal disease on diabetes: systematic review of epidemiologic observational evidence. J Clin Periodontol 40(Suppl 14):S135–S152. 10.1111/jcpe.12080 23627324 10.1111/jcpe.12080

[15] Van Dyke TE, Sheilesh D (2005) Risk factors for periodontitis. J Int Acad Periodontol 7:3–7 15736889 PMC1351013

[16] Trombelli L, Farina R, Silva CO, Tatakis DN (2018) Plaque-induced gingivitis: Case definition and diagnostic considerations. J Periodontol 89(Suppl 1):S46–S73. 10.1002/JPER.17-0576 29926936 10.1002/JPER.17-0576

[17] Baiju RMP, Peter E, Nayar BR, Varughese JM, Varghese NO (2019) Prevalence and predictors of early periodontal disease among adolescents. J Indian Soc Periodontol 23:356–361. 10.4103/jisp.jisp_512_18 31367134 10.4103/jisp.jisp_512_18 PMC6628767

[18] Helal O, Gostemeyer G, Krois J, Fawzy El Sayed K, Graetz C, Schwendicke F (2019) Predictors for tooth loss in periodontitis patients: Systematic review and meta-analysis. J Clin Periodontol 46:699–712. 10.1111/jcpe.13118 31025366 10.1111/jcpe.13118

[19] Luis HS, Luis LS, Bernardo M, Dos Santos NR (2018) Randomized controlled trial on mouth rinse and flossing efficacy on interproximal gingivitis and dental plaque. Int J Dent Hyg 16:e73–e78. 10.1111/idh.12307 28834178 10.1111/idh.12307

[20] Karkhanechi M, Chow D, Sipkin J, Sherman D, Boylan RJ, Norman RG, Craig RG, Cisneros GJ (2013) Periodontal status of adult patients treated with fixed buccal appliances and removable aligners over one year of active orthodontic therapy. Angle Orthod 83:146–151. 10.2319/031212-217.1 22725616 10.2319/031212-217.1 PMC8805524

[21] Levrini L, Mangano A, Montanari P, Margherini S, Caprioglio A, Abbate GM (2015) Periodontal health status in patients treated with the Invisalign((R)) system and fixed orthodontic appliances: A 3 months clinical and microbiological evaluation. Eur J Dent 9:404–410. 10.4103/1305-7456.163218 26430371 10.4103/1305-7456.163218 PMC4569994

[22] Haffajee AD, Socransky SS (1994) Microbial etiological agents of destructive periodontal diseases. Periodontol 200 5:78–111. 10.1111/j.1600-0757.1994.tb00020.x 10.1111/j.1600-0757.1994.tb00020.x 9673164

[23] Hellwig EKJ, Attin T (2009) Einführung in die Zahnerhaltung; Prüfungswissen Kariologie Endodontologie und Parodontologie, Germany

[24] Loesche WJ (1993) Bacterial mediators in periodontal disease. Clin Infect Dis 16(Suppl 4):S203–S210. 10.1093/clinids/16.supplement_4.s203 8324120 10.1093/clinids/16.supplement_4.s203

[25] Socransky SS, Haffajee AD, Cugini MA, Smith C, Kent RL Jr (1998) Microbial complexes in subgingival plaque. J Clin Periodontol 25:134–144. 10.1111/j.1600-051x.1998.tb02419.x 9495612 10.1111/j.1600-051x.1998.tb02419.x

[26] Ehlers V, Kasaj A, Prescher N, Willershausen B (2008) Tagungsbeitrag-MMP-8-Messung bei Patienten mit chronischer Parodontitis und Schwangerschaftsgingivitis. Deutsche Zahnarztliche Zeitschrift 63:206

[27] da Silva MK, de Carvalho ACG, Alves EHP, da Silva FRP, Pessoa LDS, Vasconcelos DFP (2017) Genetic factors and the risk of periodontitis development: findings from a systematic review composed of 13 studies of meta-analysis with 71,531 participants. Int J Dent 2017:1914073. 10.1155/2017/1914073 28529526 10.1155/2017/1914073 PMC5424192

[28] Lang NP, Tonetti MS, Suter J, Sorrell J, Duff GW, Kornman KS (2000) Effect of interleukin-1 gene polymorphisms on gingival inflammation assessed by bleeding on probing in a periodontal maintenance population. J Periodontal Res 35:102–107. 10.1034/j.1600-0765.2000.035002102.x 10863964 10.1034/j.1600-0765.2000.035002102.x

[29] de Morais EF, Pinheiro JC, Leite RB, Santos PPA, Barboza CAG, Freitas RA (2018) Matrix metalloproteinase-8 levels in periodontal disease patients: a systematic review. J Periodontal Res 53:156–163. 10.1111/jre.12495 28898418 10.1111/jre.12495

[30] Kornman KS, Crane A, Wang HY, di Giovine FS, Newman MG, Pirk FW, Wilson TG Jr, Higginbottom FL, Duff GW (1997) The interleukin-1 genotype as a severity factor in adult periodontal disease. J Clin Periodontol 24:72–77. 10.1111/j.1600-051x.1997.tb01187.x 9049801 10.1111/j.1600-051x.1997.tb01187.x

[31] Laine ML, Farre MA, Gonzalez G, van Dijk LJ, Ham AJ, Winkel EG, Crusius JB, Vandenbroucke JP, van Winkelhoff AJ, Pena AS (2001) Polymorphisms of the interleukin-1 gene family, oral microbial pathogens, and smoking in adult periodontitis. J Dent Res 80:1695–1699. 10.1177/00220345010800080301 11669477 10.1177/00220345010800080301

[32] Rams TE, Oler J, Listgarten MA, Slots J (1993) Utility of Ramfjord index teeth to assess periodontal disease progression in longitudinal studies. J Clin Periodontol 20:147–150. 10.1111/j.1600-051x.1993.tb00330.x 8436634 10.1111/j.1600-051x.1993.tb00330.x

[33] Kinney JS, Ramseier CA, Giannobile WV (2007) Oral fluid-based biomarkers of alveolar bone loss in periodontitis. Ann N Y Acad Sci 1098:230–251. 10.1196/annals.1384.028 17435132 10.1196/annals.1384.028 PMC2570328

[34] Loe H, Silness J (1963) Periodontal disease in pregnancy, prevalence and severity. Acta Odontol Scand 21:532–551 10.3109/00016356309011240 14121956

[35] Kossack C, Jost-Brinkmann PG (2005) Plaque and gingivitis reduction in patients undergoing orthodontic treatment with fixed appliances-comparison of toothbrushes and interdental cleaning aids. A 6-month clinical single-blind trial. J Orofac Orthop 66:20–38. 10.1007/s00056-005-0344-4 15711898 10.1007/s00056-005-0344-4

[36] Brunson JCR, QD (2023) Ggalluvial: Alluvial&xD; plots in & pos; ggplot2 & apos: R package version 0.12.5. https://corybrunson.github.i.o./ggalluvial/

[37] dG J (2012) Gebrauchsanleitung dentoELISA aMMP-8 Version 09. https://dentognostics.de/uploads/2012

[38] Hadzic S, Gojkov-Vukelic M, Pasic E, Jahic IM, Muharemovic A, Redzepagic-Vrazalica L, Jeleskovic A, Nakas E (2022) Evaluation of periodontal changes in patients before, during, and after a fixed orthodontic therapy. Mater Sociomed 34:121–125. 10.5455/msm.2022.34.121-125 36199847 10.5455/msm.2022.34.121-125 PMC9478536

[39] Peron PF, Wehrbein H, Mundethu A, Schmidtmann I, Erbe C (2024) Clinical parameters and inflammatory biomarkers among patients with multibracket appliances: a prospective clinical trial. BMC Oral Health 24:308. 10.1186/s12903-024-03995-3 38443926 10.1186/s12903-024-03995-3 PMC10913366

[40] Lee A, Ghaname CB, Braun TM, Sugai JV, Teles RP, Loesche WJ, Kornman KS, Giannobile WV, Kinney JS (2012) Bacterial and salivary biomarkers predict the gingival inflammatory profile. J Periodontol 83:79–89. 10.1902/jop.2011.110060 21563952 10.1902/jop.2011.110060

[41] Loomer PM (2004) Microbiological diagnostic testing in the treatment of periodontal diseases. Periodontology 200 34:49–56. 10.1046/j.0906-6713.2002.003424.x 10.1046/j.0906-6713.2002.003424.x 14717855

[42] Socransky SS, Haffajee AD (2002) Dental biofilms: difficult therapeutic targets. Periodontology 2000(28):12–55. 10.1034/j.1600-0757.2002.280102.x 10.1034/j.1600-0757.2002.280102.x 12013340

[43] Lombardo L, Palone M, Scapoli L, Siciliani G, Carinci F (2021) Short-term variation in the subgingival microbiota in two groups of patients treated with clear aligners and vestibular fixed appliances: A longitudinal study. Orthod Craniofac Res 24:251–260. 10.1111/ocr.12427 32965768 10.1111/ocr.12427

[44] Sfondrini MF, Butera A, Di Michele P et al (2021) Microbiological changes during orthodontic aligner therapy: a prospective clinical trial. 10.3390/app11156758

[45] Ferlazzo N, Curro M, Zinellu A, Caccamo D, Isola G, Ventura V, Carru C, Matarese G, Ientile R (2017) Influence of MTHFR genetic background on p16 and MGMT methylation in oral squamous cell cancer. Int J Mol Sci 18:. 10.3390/ijms18040724 10.3390/ijms18040724 PMC5412310 28353639

[46] Guo R, Lin Y, Zheng Y, Li W (2017) The microbial changes in subgingival plaques of orthodontic patients: a systematic review and meta-analysis of clinical trials. BMC Oral Health 17:90. 10.1186/s12903-017-0378-1 28576147 10.1186/s12903-017-0378-1 PMC5455174

[47] Kim SH, Choi DS, Jang I, Cha BK, Jost-Brinkmann PG, Song JS (2012) Microbiologic changes in subgingival plaque before and during the early period of orthodontic treatment. Angle Orthod 82:254–260. 10.2319/030311-156.1 21827233 10.2319/030311-156.1 PMC8867936

[48] Pan S, Liu Y, Si Y, Zhang Q, Wang L, Liu J, Wang C, Xiao S (2017) Prevalence of fimA genotypes of Porphyromonas gingivalis in adolescent orthodontic patients. PLoS ONE 12:e0188420. 10.1371/journal.pone.0188420 29176857 10.1371/journal.pone.0188420 PMC5703466

[49] Sun F, Ahmed A, Wang L, Dong M, Niu W (2018) Comparison of oral microbiota in orthodontic patients and healthy individuals. Microb Pathog 123:473–477. 10.1016/j.micpath.2018.08.011 30096429 10.1016/j.micpath.2018.08.011

[50] Gujar AN, Al-Hazmi A, Raj AT, Patil S (2020) Microbial profile in different orthodontic appliances by checkerboard DNA-DNA hybridization: An in-vivo study. Am J Orthod Dentofacial Orthop 157:49–58. 10.1016/j.ajodo.2019.01.026 31901280 10.1016/j.ajodo.2019.01.026

[51] Diamantikipioti A, Gusberti FA, Lang NP (1987) Clinical and microbiological effects of fixed orthodontic appliances. J Clin Periodontol 14:326–333. 10.1111/j.1600-051X.1987.tb00979.x 3509967 10.1111/j.1600-051x.1987.tb00979.x

[52] Naranjo AA, Trivino ML, Jaramillo A, Betancourth M, Botero JE (2006) Changes in the subgingival microbiota and periodontal parameters before and 3 months after bracket placement. Am J Orthod Dentofacial Orthop 130(275):e17-22. 10.1016/j.ajodo.2005.10.022 10.1016/j.ajodo.2005.10.022 16979483

[53] Ristic M, Vlahovic Svabic M, Sasic M, Zelic O (2008) Effects of fixed orthodontic appliances on subgingival microflora. Int J Dent Hyg 6:129–136. 10.1111/j.1601-5037.2008.00283.x 18412726 10.1111/j.1601-5037.2008.00283.x

[54] Thornberg MJ, Riolo CS, Bayirli B, Riolo ML, Van Tubergen EA, Kulbersh R (2009) Periodontal pathogen levels in adolescents before, during, and after fixed orthodontic appliance therapy. Am J Orthod Dentofacial Orthop 135:95–98. 10.1016/j.ajodo.2007.02.057 19121507 10.1016/j.ajodo.2007.02.057

[55] Erbe C, Hornikel S, Schmidtmann I, Wehrbein H (2011) Quantity and distribution of plaque in orthodontic patients treated with molar bands. J Orofac Orthop 72:13–20. 10.1007/s00056-010-0001-4 21484542 10.1007/s00056-010-0001-4

[56] Izadi Borujeni S, Mayer M, Eickholz P (2015) Activated matrix metalloproteinase-8 in saliva as diagnostic test for periodontal disease? A case-control study. Med Microbiol Immunol 204:665–672. 10.1007/s00430-015-0413-2 25841875 10.1007/s00430-015-0413-2

[57] Ingman T, Apajalahti S, Mantyla P, Savolainen P, Sorsa T (2005) Matrix metalloproteinase-1 and -8 in gingival crevicular fluid during orthodontic tooth movement: a pilot study during 1 month of follow-up after fixed appliance activation. Eur J Orthod 27:202–207. 10.1093/ejo/cjh097 15817630 10.1093/ejo/cjh097

[58] Apajalahti S, Sorsa T, Railavo S, Ingman T (2003) The in vivo levels of matrix metalloproteinase-1 and -8 in gingival crevicular fluid during initial orthodontic tooth movement. J Dent Res 82:1018–1022. 10.1177/154405910308201216 14630906 10.1177/154405910308201216

[59] Canavarro C, Teles RP, Capelli Junior J (2013) Matrix metalloproteinases -1, -2, -3, -7, -8, -12, and -13 in gingival crevicular fluid during orthodontic tooth movement: a longitudinal randomized split-mouth study. Eur J Orthod 35:652–658. 10.1093/ejo/cjs053 22989715 10.1093/ejo/cjs053

[60] Almeida RC, Capelli J Jr, Teles RP (2015) Levels of gingival crevicular fluid matrix metalloproteinases in periodontally compromised teeth under orthodontic forces. Angle Orthod 85:1009–1014. 10.2319/101714-744.1 25751014 10.2319/101714-744.1 PMC8612058

[61] Bildt MM, Bloemen M, Kuijpers-Jagtman AM, Von den Hoff JW (2009) Matrix metalloproteinases and tissue inhibitors of metalloproteinases in gingival crevicular fluid during orthodontic tooth movement. Eur J Orthod 31:529–535. 10.1093/ejo/cjn127 19299245 10.1093/ejo/cjn127

[62] Pani P, Tsilioni I, McGlennen R, Brown CA, Hawley CE, Theoharides TC, Papathanasiou E (2021) IL-1B(3954) polymorphism and red complex bacteria increase IL-1beta (GCF) levels in periodontitis. J Periodontal Res 56:501–511. 10.1111/jre.12850 33638191 10.1111/jre.12850 PMC8799375

[63] Teles R, Sakellari D, Teles F, Konstantinidis A, Kent R, Socransky S, Haffajee A (2010) Relationships among gingival crevicular fluid biomarkers, clinical parameters of periodontal disease, and the subgingival microbiota. J Periodontol 81:89–98. 10.1902/jop.2009.090397 20059421 10.1902/jop.2009.090397 PMC2805280

[64] Shokeen B, Viloria E, Duong E, Rizvi M, Murillo G, Mullen J, Shi B, Dinis M, Li H, Tran NC, Lux R, Wu T (2022) The impact of fixed orthodontic appliances and clear aligners on the oral microbiome and the association with clinical parameters: A longitudinal comparative study. Am J Orthod Dentofacial Orthop 161:e475–e485. 10.1016/j.ajodo.2021.10.015 35248417 10.1016/j.ajodo.2021.10.015

[65] Al-Mutairi MA, Al-Salamah L, Nouri LA, Al-Marshedy BS, Al-Harbi NH, Al-Harabi EA, Al-Dosere HA, Tashkandi FS, Al-Shabib ZM, Altalhi AM (2024) Microbial changes in the periodontal environment due to orthodontic appliances: a review. Cureus 16:e64396. 10.7759/cureus.64396 39130947 10.7759/cureus.64396 PMC11317031

[66] Barlattani A Jr, Mampieri G, Ottria L, Bollero P (2009) Invisalign treatment in periodondal patient: case report. Oral Implantol 2:35 PMC3415342 23285373

[67] Boyd RL, Waskalic V (2001) Three-dimensional diagnosis andorthodontic treatment of complex malocclusions with the invisalign appliance. Semin Orthod 7:274–293

[68] Lu H, Tang H, Zhou T, Kang N (2018) Assessment of the periodontal health status in patients undergoing orthodontic treatment with fixed appliances and Invisalign system: A meta-analysis. Medicine (Baltimore) 97:e0248. 10.1097/MD.0000000000010248 29595680 10.1097/MD.0000000000010248 PMC5895427

[69] Partouche AJD, Castro F, Baptista AS, Costa LG, Fernandes JCH, Fernandes GVO (2022) Effects of multibracket orthodontic treatment versus clear aligners on periodontal health: an integrative review. Dent J (Basel) 10: 10.3390/dj10100177 10.3390/dj10100177 PMC9600623 36285987

[70] Wu Y, Cao L, Cong J (2020) The periodontal status of removable appliances vs fixed appliances: A comparative meta-analysis. Medicine (Baltimore) 99:e23165. 10.1097/MD.0000000000023165 33327234 10.1097/MD.0000000000023165 PMC7738141

[71] Di Spirito F, D’Ambrosio F, Cannata D, D'Anto V, Giordano F, Martina S (2023) Impact of clear aligners versus fixed appliances on periodontal status of patients undergoing orthodontic treatment: a systematic review of systematic reviews. Healthcare (Basel) 11:. 10.3390/healthcare11091340 10.3390/healthcare11091340 PMC10178428 37174882

[72] Rossini G, Parrini S, Castroflorio T, Deregibus A, Debernardi CL (2015) Periodontal health during clear aligners treatment: a systematic review. Eur J Orthod 37:539–543. 10.1093/ejo/cju083 25548145 10.1093/ejo/cju083

[73] Zhao R, Huang R, Long H, Li Y, Gao M, Lai W (2020) The dynamics of the oral microbiome and oral health among patients receiving clear aligner orthodontic treatment. Oral Dis 26:473–483. 10.1111/odi.13175 31418980 10.1111/odi.13175

[74] Favero R, Libralato L, Balestro F, Volpato A, Favero L (2023) Edge level of aligners and periodontal health: a clinical perspective study in young patients. Dental Press J Orthod 28:e2321124. 10.1590/2177-6709.28.1.e2321124.oar 37075415 10.1590/2177-6709.28.1.e2321124.oar PMC10108582

[75] Traversa F, Chavanne P, Mah J (2024) Biomechanics of clear aligner therapy: Assessing the influence of tooth position and flat trimline height in translational movements. Orthod Craniofac Res:. 10.1111/ocr.12796 10.1111/ocr.12796 PMC11701961 38685814

[76] Chen W, Chen J, Bai D, Wang P, Shu R (2024) Effects of clear aligners and traditional removable appliances on oral microbiome in mixed dentition: a comparative study. BMC Oral Health 24:1276. 10.1186/s12903-024-05063-2 39448993 10.1186/s12903-024-05063-2 PMC11515335

[77] Arendorf T, Addy M (1985) Candidal carriage and plaque distribution before, during and after removable orthodontic appliance therapy. J Clin Periodontol 12:360–368. 10.1111/j.1600-051x.1985.tb00926.x 3859496 10.1111/j.1600-051x.1985.tb00926.x

[78] Petti S, Barbato E, Simonetti D’Arca A (1997) Effect of orthodontic therapy with fixed and removable appliances on oral microbiota: a six-month longitudinal study. New Microbiol 20:55–62 9037669

